# Micronucleus Formation Induced by Glyphosate and Glyphosate-Based Herbicides in Human Peripheral White Blood Cells

**DOI:** 10.3389/fpubh.2021.639143

**Published:** 2021-05-24

**Authors:** Károly Nagy, Roba Argaw Tessema, István Szász, Tamara Smeirat, Alaa Al Rajo, Balázs Ádám

**Affiliations:** ^1^Department of Public Health and Epidemiology, Faculty of Medicine, University of Debrecen, Debrecen, Hungary; ^2^Doctoral School of Health Sciences, University of Debrecen, Debrecen, Hungary; ^3^College of Medicine and Health Sciences, Institute of Public Health, United Arab Emirates University, Al Ain, United Arab Emirates

**Keywords:** glyphosate, formulation, GBHs, micronucleus, cytotoxicity, genotoxicity

## Abstract

Glyphosate is the most commonly used herbicide around the world, which led to its accumulation in the environment and consequent ubiquitous human exposure. Glyphosate is marketed in numerous glyphosate-based herbicide formulations (GBHs) that include co-formulants to enhance herbicidal effect of the active ingredient, but are declared as inert substances. However, these other ingredients can have biologic activity on their own and may interact with the glyphosate in synergistic toxicity. In this study, we focused to compare the cytogenetic effect of the active ingredient glyphosate and three marketed GBHs (Roundup Mega, Fozat 480, and Glyfos) by investigating cytotoxicity with fluorescent co-labeling and WST-1 cell viability assay as well as genotoxicity with cytokinesis block micronucleus assay in isolated human mononuclear white blood cells. Glyphosate had no notable cytotoxic activity over the tested concentration range (0–10,000 μM), whereas all the selected GBHs induced significant cell death from 1,000 μM regardless of metabolic activation (S9). Micronucleus (MN) formation induced by glyphosate and its formulations at sub-cytotoxic concentrations (0–100 μM) exhibited a diverse pattern. Glyphosate caused statistically significant increase of MN frequency at the highest concentration (100 μM) after 20-h exposure. Contrarily, Roundup Mega exerted a significant genotoxic effect at 100 μM both after 4- and 20-h exposures; moreover, Glyfos and Fozat 480 also resulted in a statistically significant increase of MN frequency from the concentration of 10 μM after 4-h and 20-h treatment, respectively. The presence of S9 had no effect on MN formation induced by either glyphosate or GBHs. The differences observed in the cytotoxic and genotoxic pattern between the active principle and formulations confirm the previous concept that the presence of co-formulants in the formulations or the interaction of them with the active ingredient is responsible for the increased toxicity of herbicide products, and draw attention to the fact that GBHs are still currently in use, the toxicity of which rivals that of POEA-containing formulations (e.g., Glyfos) already banned in Europe. Hence, it is advisable to subject them to further comprehensive toxicological screening to assess the true health risks of exposed individuals, and to reconsider their free availability to any users.

## Introduction

Our consumer society has reached the point where a chemical marketed in the United States in the 1970s and declared harmless for decades is virtually everywhere: in natural waters, meat, wine, beer, and even in the urine of many of us (1, 2). This chemical is one of the pesticides with the highest sales volume on the market, glyphosate. It is the active ingredient of numerous glyphosate-based herbicides (GBHs), and due to its use in small gardens, it is perhaps the best-known agrochemical. To date, there is no substitute that would produce the same efficacy as glyphosate, and we have to expect a large rate of crop losses worldwide if it is ever banned. Nevertheless, there is a real civil rights movement against glyphosate in the United States today, in the courts, because the International Agency for Research on Cancer (IARC) classified glyphosate as a “probable human carcinogen” in March 2015 (3). As a result, by the end of 2019, more than 40,000 lawsuits had been filed by American citizens suffering from non-Hodgkin lymphoma (NHL) and presumably exposed to glyphosate-based herbicides, three of whom have gone to trial and each won the lawsuit (4). At the same time, surprising developments have come to light. Neither the European Food Safety Authority (EFSA) nor the US Environmental Protection Agency (EPA) did find sufficient evidence about the carcinogenic potential of the herbicide in October 2015 and September 2016, respectively (5, 6). The discrepancy between the findings of each agency was attributed to the facts that EFSA and EPA relied mostly on unpublished studies funded by herbicide manufacturers, while IARC did not. In addition, IARC placed heavy weight on the cocktail effect of formulated GBHs (i.e., when glyphosate is used with another chemical in a formulation) whereas EFSA and EPA did not (4, 7). In October 2017, the European Parliament supported the withdrawal of glyphosate within 5 years, but 2 months later the European Commission voted to re-authorize the active substance in the EU for 5 more years (8). This decision raises deep concern in the light of the findings of recent meta-analytic studies that, combining results of numerous epidemiological investigations, have identified a compelling link between real-life glyphosate exposure and NHL (9, 10).

In reality, almost no one is exposed to glyphosate as an active substance alone, but rather to complete GBHs, which contain various other ingredients labeled as “adjuvants“ or “co-formulants” that are aimed to improve the herbicidal efficacy of glyphosate, but are defined as inerts (11). Usually, the identity and concentration of supposedly inert co-formulants in GBHs are not disclosed on product labels, material and safety data sheets, or in any publicly accessible documentation of the pesticide products because they constitute business secrets (12). Therefore, the composition of numerous GBHs is unknown, that makes it very difficult for scientists to assess the health risks of certain adjuvants or the combined effects of different ingredients in GBHs. Notwithstanding, more and more evidence has emerged that certain adjuvants, such as polyethoxylated tallow amines (POEAs), are more toxic than glyphosate alone (13, 14), and/or increase the toxicity of the active substance by allowing it to penetrate plant, but also animal and human, cells more easily, increasing formulations' toxicity (15, 16). As a result of this recognition, GBHs containing these adjuvants are progressively being phased out and replaced by a new generation of co-formulants on the European market, but not in the US. The toxic potential of GBHs formulated with non-POEA adjuvants is reported to be lower than that with POEAs (17); nevertheless, there is still a considerable knowledge gap in the systematic assessment of chronic health risks posed by the exposure to commercially available GBHs with still highly variable composition.

The IARC classification for glyphosate was partly based on strong evidence that glyphosate or GBHs are able to induce genotoxicity, recognized first step in carcinogenesis, in human cells *in vitro* and in experimental animals (3). Recently, comprehensive reviews summarizing animal carcinogenicity data and data from multiple *in vitro* and *in vivo* genotoxicity assays have further supported the IARC statement (18, 19). DNA damage indicated by DNA strand breaks as a marker of genotoxicity was observed as a result of exposure to glyphosate as an active ingredient in various human cell types *in vitro* (20–25) as well as in mice (26) and fish (23, 27, 28) *in vivo*. GBHs induced DNA damage in human liver HepG2 cells (29), buccal carcinoma cells (TR146) (21) and peripheral blood mononuclear cells (30) *in vitro* as well as in mice *in vivo* (26). Another marker which can help to assess the genotoxic properties of xenobiotics is the presence of micronuclei (MN) that indicates clastogenic events in eukaryotic cells. MN can be formed from acentric chromosomal fragments or whole chromosomes left behind during mitotic cellular division, and can be detected by the cytokinesis-block micronucleus (CBMN) assay, which is a standardized, sensitive and simple laboratory technique to evaluate genomic damage in isolated cells (31). MN was successfully detected as a biomarker in several human biomonitoring studies of pesticide-exposed individuals (32–34) including GBH-exposed humans (35). A large-scale systematic meta-analytical review involving 93, mainly non-human, experimental studies has been recently carried out to analyze the relationship between exposure to glyphosate or GBHs and the formation of MN. The review concluded in general that both the active ingredient and the formulations increase the frequency of MN in tested organisms (36). Examples include, but are not limited to, studies reporting MN formation after exposure to glyphosate as an active ingredient in isolated human lymphocytes (22, 37) and HepG2 cells (38) *in vitro* and in mice (26) *in vivo*. GBHs were also able to induce MN formation in polychromatic erythrocytes of mice (26, 39) *in vivo*.

Although genotoxic and mutagenic effects of glyphosate and GBHs have already been extensively studied with various methods, there is still insufficient evidence on the possible complex interactions between ingredients in GBHs, and the effect of glyphosate-based formulations on the induction of MN formation in human cells has not been investigated so far. As a continuation of our previous study that focused on the comparative analysis of primary DNA damage induced by three marketed GBHs with different composition and the active ingredient glyphosate, herein, we compare the clastogenic activity of the same GBHs to glyphosate as well as to each other in human mononuclear white blood cells (HMWB) *in vitro* using the CBMN assay.

## Materials and Methods

### Chemicals

Analytical-grade glyphosate (N-(phosphonomethyl) glycine, CAS No: 1071-83-6) was purchased from VWR International Kft (Debrecen, Hungary). Samples of three GBHs, namely

- Roundup Mega containing 551 g/L or 42% (w/w) potassium salt of glyphosate (CAS No: 70901-12-1; equivalent to 450 g/L glyphosate) and 7% (w/w) ethoxylated etheralkylamine (CAS No: 68478-96-6);
- Fozat 480 containing 480 g/L or 41% (w/w) isopropylammonium salt of glyphosate (CAS No: 38641-94-0; equivalent to 360 g/L glyphosate) and <5% (w/w) hygroscopic substances;
- Glyfos containing 480 g/L or 42% (w/w) isopropylammonium salt of glyphosate (equivalent to 360 g/L glyphosate) and 9% (w/w) polyethoxylated tallow amine (CAS No: 61791-26-2);

were kindly provided by pesticide applicators. Composition data for each formulation were retrieved from the material safety data sheets (MSDS). Chemicals used for the assays and human liver-derived metabolic activation system (S9 fraction) were obtained from Sigma-Aldrich Chemie GmbH (Heidelberg, Germany). Cell culture medium and its supplements were obtained from Biowest (Nuaillè, France). The acetomethoxy derivative of calcein (Calcein AM) and propidium iodide (PIO) fluorescent dyes were purchased from Biotium (Hayward, CA, USA). Heparin-containing vacutainers were purchased from BD Vacutainer Systems (Plymouth, UK).

### Cell Cultures

Human peripheral whole blood samples were obtained by venipuncture and collected into heparin-containing vacutainer tubes from three non-smoking, healthy volunteers (males, aged 20–40 years) without known previous contact with pesticides. Cultures were prepared within 1-h of phlebotomy. 0.3 mL heparinized whole blood was added to 4.7 mL RPMI-1640 complete medium supplemented with 10% FBS, 100 U/mL penicillin, 100 μg/mL streptomycin, 250 ng/mL amphotericin and 1.5% phytohemagglutinin. Whole blood samples were cultured for 48-h before treatment. All donors signed the informed consent. The study was approved by the Hungarian Ethical Committee for Medical Research (document 147-5/2019/EÜIG) and was performed in accordance with the ethical standards laid down in the 2013 Declaration of Helsinki.

### Cell Treatment

The cells were exposed to glyphosate at final concentrations of 0.1, 1, 10, 100, 1,000, 10,000 μM and to three GBHs at the same glyphosate-equivalent final concentrations. The concentrations of GBHs are referred to as glyphosate equivalent concentrations in this study.

The stock solutions and the dilution series were made in phosphate-buffered saline (PBS) and adjusted to pH 7.2. Aliquots of different concentrations of glyphosate and GBH solutions, as well as PBS as negative control and 1.3 μM bleomycin sulfate (BLEO) as a positive control, were added to the cell cultures and incubated for 4- and 20-h at 37°C. The PBS content was always <10% (v/v) in the cell culture medium.

All the experiments were conducted in the presence and absence of S9 fraction. Hundred microliter of the working S9 mix containing 10% (v/v) of S9 fraction was composed of 8 mM MgCl_2_, 33 mM KCl, 100 mM sodium phosphate buffer pH 7.4, 5 mM glucose-6-phosphate, and 4 mM NADP was added to the S9+ samples.

### Calcein AM and Propidium Iodide Cell Viability Assay

After treatment and removal of erythrocytes by hypotonic (0.075 M KCl) lysis, Calcein AM and PIO fluorescent dyes were used to co-label the HMWB cells. Calcein AM is a non-polar compound that passively crosses the plasma membrane of living cells, where it is cleaved by intracellular esterases to reveal a very polar derivative of fluorescein (calcein) that remains trapped in the cytoplasm. PIO is a DNA intercalating dye, which is able to permeate membranes of dead and dying cells but cannot penetrate plasma membranes of live healthy cells.

Both fluorescent dyes were dissolved in PBS (pH 7.2) to a final concentration of 2 μM each. Two hundred microliter of this working solution were added to the cell pellets (1 × 10^5^ cells) and incubated for 30 min at 4°C, protected from light. The labeled cells were washed and re-suspended in ice-cold PBS buffer. Forty microliter of the cell suspension was put on a microscope slide for immediate microscopic examination.

FITC filter for Calcein AM and TRITC filter for PIO was applied to excite the co-labeled cells. Survival rate was determined by visual examination of 10 randomly selected non-overlapping fields per slide. Each field contained 10 to 30 images.

### WST-1 Cell Viability Assay

The WST-1 cell proliferation reagent was applied according to the manufacturer's protocol. Before treatment, HMWB cells were separated from erythrocytes by density-gradient centrifugation over Histopaque-1077 gradient to avoid interference caused by residual hemoglobin during absorbance measurement that would have resulted from using hypotonic lysis of erythrocytes. The buffy coat was then aspirated and re-suspended in RPMI 1640 medium containing 10% fetal calf serum (FCS). HMWB cells were seeded in Eppendorf tubes at a cell number of 1 × 10^5^ and were treated with the test chemicals as described in the Cell treatment section. Following treatment, samples were centrifuged, the supernatant was discarded, and HMWB cells were resuspended in 100 μL RPMI-1640 complete medium supplemented with 10% FBS, 100 U/mL penicillin, 100 μg/mL streptomycin and 250 ng/mL amphotericin. Samples were then transferred to flat-bottomed 96-well plate, and 10 μL of WST-1 was added directly to the culture in each well. The cells were incubated for 3-h at 37°C. Absorbance at 440 nm was measured using an Epoch™ Microplate Spectrophotometer (BioTek Instruments, Winooski, VT, USA). The reference absorbance was set at 700 nm. Cell viability was calculated by dividing the absorbance of the treated cells by that of the vehicle-treated (PBS) control cells (considered 100%).

### Cytokinesis-Block Micronucleus Assay

CBMN assay was carried out following the previously reported standardized protocol (OECD guideline) (40) with slight modifications (41). After 4-h treatment with glyphosate alone and with the three GBHs, whole blood cells were centrifuged, the supernatant was removed, and the cells were resuspended in 3 μg/mL cytochalasin B containing medium. In case of 20-h treatment, cytochalasin B was added in parallel with the addition of the test chemicals. Following 20-h incubation, cells were harvested for slide preparation. Whole blood samples were centrifuged and resuspended in hypotonic (0.075 M) KCl solution then fixed in cold fixative (methanol:acetic acid 5:1) for 30 min at room temperature. The latter step was repeated twice to completely remove the erythrocytes.

Cell suspensions were carefully dropped onto clean wet slides to disperse the cells. Slides were air dried, stained with 3% Giemsa in distilled water and mounted in Eukitt.

Giemsa-stained slides were coded and analyzed blindly by two scorers under a magnification of 400. Proliferation index (PI) was determined by counting at least 500 cells with one, two or more than two nuclei. The PI was calculated according to the formula: PI = M_1_ + 2M_2_ + 3M_multi_/n, where M_1_ to M_multi_ represent the number of cells with one to multiple (more than 2) nuclei and n is the number of cells scored. In total, 2,000 binucleated cells (1,000 per slide) were scored from each experimental point. The MN frequency was calculated as the ratio of the number of binucleated cells with micronuclei (BNMN) to the binucleated cells. The identification of MN was performed according to the criteria described by Fenech et al. (42).

### Data Analysis

Experiments were independently performed three times from three different donors. Cell viability was expressed as the mean proportions of living cells from repeated experiments. The rate of cell viability, the frequency of binucleated cells with micronuclei and the proliferation index induced by various concentrations of the test chemicals in repeated experiments were statistically compared to that of untreated cells using ANOVA with Dunnett's *post hoc* test. The same statistical test was used to analyze the effect of metabolic activation by comparing the micronucleus frequency and proliferation index of S9-treated and S9-untreated samples at each exposure concentration. Statistically significant difference was accepted at 5% significance level.

## Results

### Cell Viability

**Figure 1:**
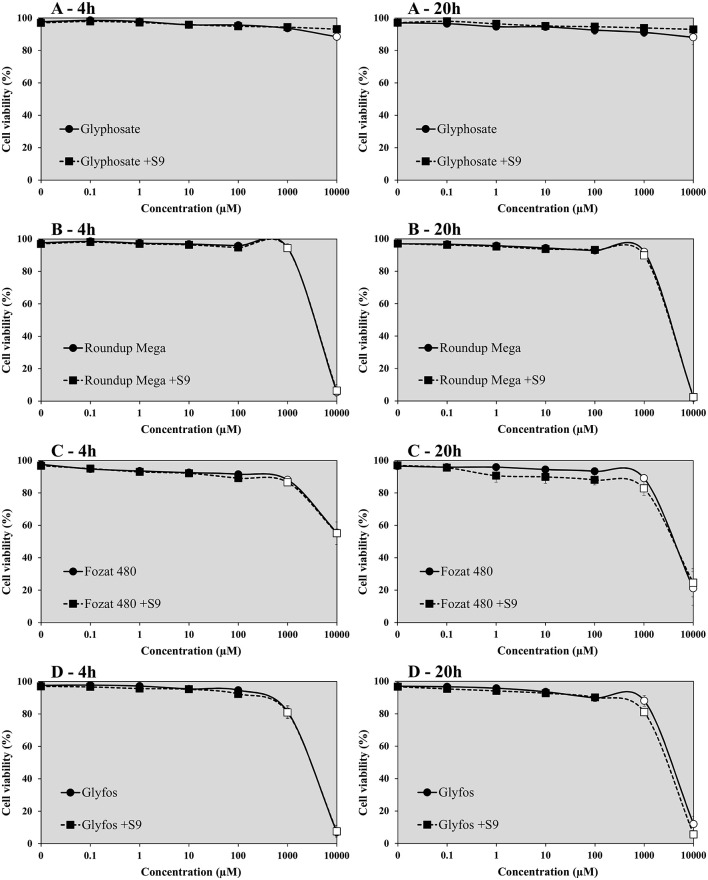
Effect of 4- and 20-h exposure to increasing concentrations of glyphosate **(A)**, Roundup Mega **(B)**, Fozat 480 **(C)**, and Glyfos **(D)** on cell viability in the absence and presence (+S9) of metabolic activation system detected by fluorescent co-labeling. The data points indicate the means ± standard error of the mean (SEM) of three repeated experiments. Statistically significant decrease of cell viability, indicated by empty data points, was determined by comparing the values induced by various doses of glyphosate or GBHs to the background level of untreated cells by ANOVA with Dunnett's *post hoc* test.

**Figure 2:**
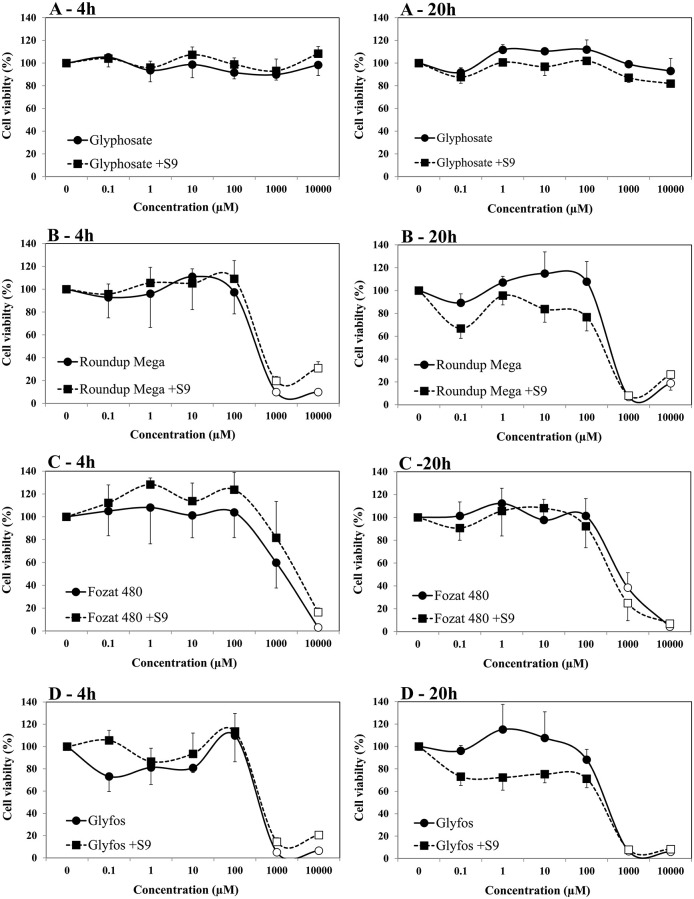
Effect of 4- and 20-h exposure to increasing concentrations of glyphosate **(A)**, Roundup Mega **(B)**, Fozat 480 **(C)**, and Glyfos **(D)** on cell viability in the absence and presence (+S9) of metabolic activation system detected by WST-1 cell viability assay. The data points indicate the means ± standard error of the mean (SEM) of three repeated experiments. Statistically significant decrease of cell viability, indicated by empty data points, was determined by comparing the values induced by various doses of glyphosate or GBHs to the background level of untreated cells by ANOVA with Dunnett's *post hoc* test.

The viability of HMWB cells treated with glyphosate alone for 4- and 20-h was found to be over 80% in the absence and presence of S9 over the entire concentration range in both cell viability assays (Figures 1, 2). A slight but statistically significant decrease of cell viability could be noticed only at 10,000 μM without S9 treatment in the fluorescent co-labeling assay at 4- and 20-h exposure, too. In contrast to the active ingredient, all the three GBHs induced a significant decrease in the proportion of living cells from 1,000 μM regardless of metabolic activation, which was more evident when using WST-1 cell viability assay. It detected significantly higher cell death at 1,000 μM concentration of the formulations compared to the fluorescent co-labeling assay.

### Micronucleus Induction

In line with the OECD Test Guideline for the *in vitro* micronucleus assay suggesting that concentrations that induce cytotoxicity >55 ± 5% should be excluded from genotoxicity testing (40), CBMN assays were carried out in a previously determined, sub-cytotoxic concentration range of the tested compounds, because cytotoxic processes, especially apoptosis, could potentially act as confounders in genotoxicity assays. As GBHs from 1,000 μM were able to be induce statistically confirmed cell death in the cytotoxicity assays, only concentrations of 0.1, 1, 10, and 100 μM were investigated in the CBMN assay.

**Figure 3:**
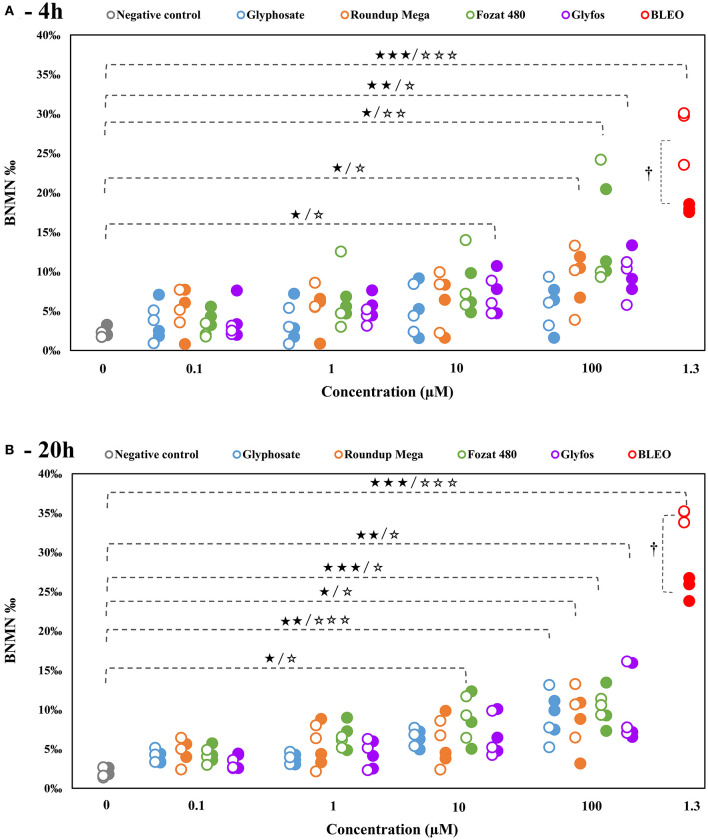
Frequency of binucleated cells with micronuclei (BNMN %) induced by 4-h **(A)** and 20-h **(B)** exposure to sub-cytotoxic concentrations of glyphosate and GBHs without (°) and with (•) metabolic activation (S9) in human mononuclear white blood cells, detected by cytokinesis-block micronucleus assay. Data points are results of individual experiments. Statistically significant (*p < 0.05, **p < 0.01, ***p < 0.001) increase was determined by comparing the frequency of binucleated cells with micronuclei induced by various doses of test chemicals to the background level of untreated cells by ANOVA with Dunnett's *post hoc* test. The same test was used to detect statistically significant (†*p* < 0.05) difference in the frequency of binucleated cells with micronuclei between S9-treated and S9-untreated cells induced by the same concentration of test chemicals.

Glyphosate alone did not cause a statistically significant increase of MN frequency except at the highest concentration (100 μM) after 20-h exposure in the absence (8.69% ± 2.34%, *p* < 0.01) and presence (9.49% ± 1.07%, *p* < 0.001) of S9, as well. By contrast, all the three GBHs induced significant increase of MN frequency at 100 μM both after 4-h with (Roundup Mega: 9.11% ± 2.76%, *p* < 0.05; Fozat 480: 14.50% ± 4.84%, *p* < 0.05; Glyfos: 9.11% ± 1.69%, *p* < 0.01) and without (Roundup Mega: 9.67% ± 1.54%, *p* < 0.05; Fozat 480: 13.93% ± 3.27%, *p* < 0.01; Glyfos: 10.01% ± 1.67%, p < 0.05) metabolic activation, as well as after 20-h in the absence (Roundup Mega: 10.12% ± 1.98%, *p* < 0.05; Fozat 480: 10.43% ± 0.59%, *p* < 0.001; Glyfos: 10.30% ± 2.92%, p < 0.01) and presence (Roundup Mega: 7.62% ± 2.31%, *p* < 0.05; Fozat 480: 10.00% ± 1.81%, *p* < 0.05; Glyfos: 9.87% ± 3.04%, p < 0.05) of S9. Moreover, 4-h treatment with Glyfos (without S9: 6.52% ± 1.22%, *p* < 0.05; with S9: 7.72% ± 1.73%, *p* < 0.05) and 20-h treatment with Fozat 480 (without S9: 9.13% ± 1.52%, *p* < 0.01; with S9: 8.59% ± 2.11%, *p* < 0.05) at the concentration of 10 μM also resulted in a significant increase of binucleated cells with micronuclei (Figure 3, Supplementary Table 1).

The presence of metabolic enzymes did not significantly alter MN frequency induced either by glyphosate alone or by the GBHs.

**Figure 4:**
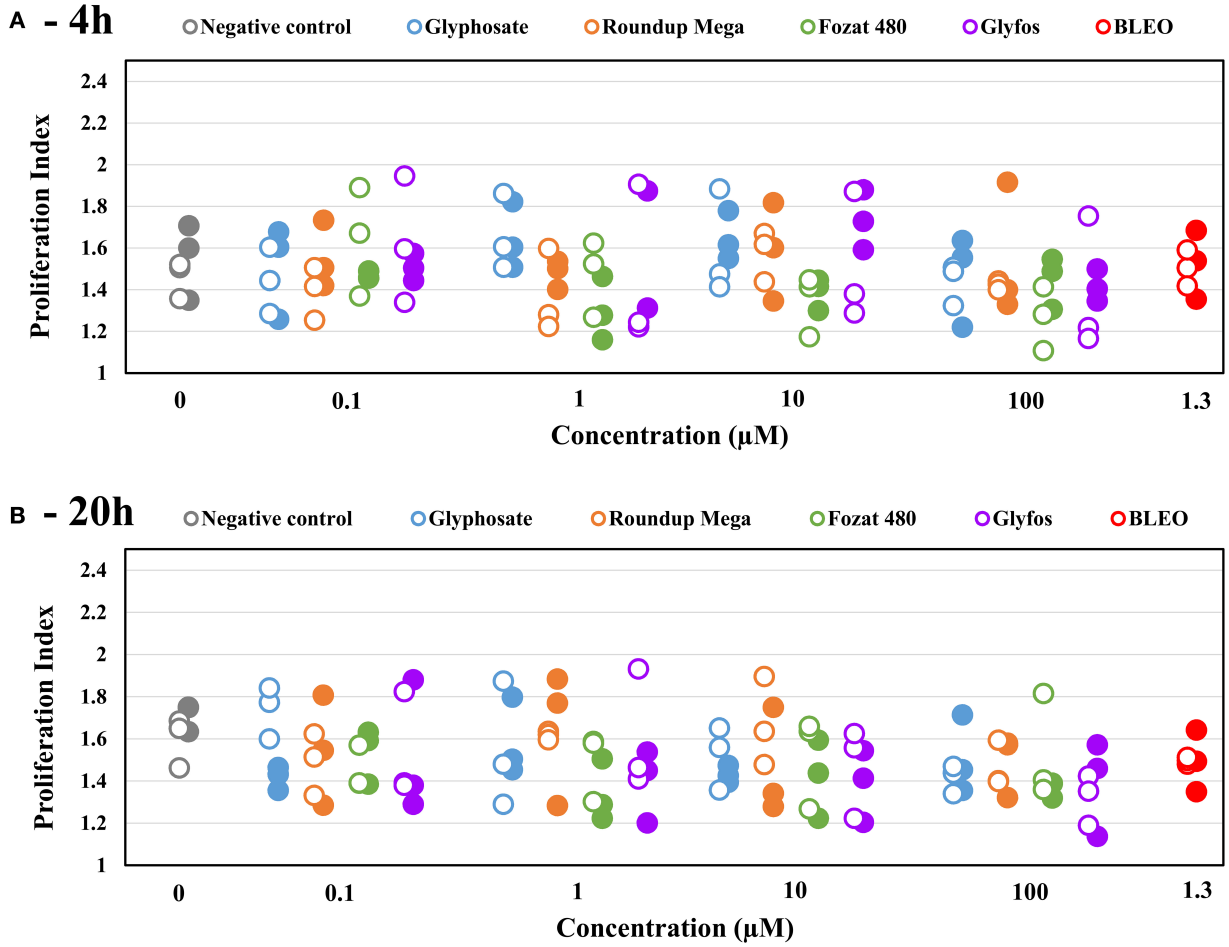
Proliferation index (PI) induced by 4-h **(A)** and 20-h **(B)** exposure to sub-cytotoxic concentrations of glyphosate and GBHs without (◦) and with (•) metabolic activation (S9) in human mononuclear white blood cells, detected by cytokinesis-block micronucleus assay. Data points are results of individual experiments. Statistically significant change was determined by comparing the proliferation index induced by various doses of test chemicals to the background level of untreated cells by ANOVA with Dunnett's *post hoc* test. The same test was used to detect statistically significant difference in the proliferation index between S9-treated and S9-untreated cells induced by the same concentration of test chemicals.

The proliferation index did not show statistically significant changes with increasing concentrations of the test chemicals at both exposure times, regardless of the presence of the metabolic enzyme system (Figure 4, Supplementary Table 2).

## Discussion

This work is the first study investigating the MN inducing ability of three GBHs along with glyphosate in isolated human cells *in vitro*. To examine whether potential metabolites of the selected herbicides can cause damage to cells, human liver-derived metabolic enzyme system (S9) was also applied. Two of the examined GBHs, Roundup Mega and Fozat 480, are permitted in Hungary and in the EU, while the POEA-containing Glyfos was withdrawn from the Hungarian market in 2017 (43).

Our data indicate that glyphosate alone could not considerably decrease the viability of HMWB cells up to 10,000 μM both after 4- and 20-h exposure regardless of metabolic activation, which is in line with our previous observations (44), and also with findings of Wozniak (30), but contradicts results of De Almeida's study, in which pure glyphosate induced a significant reduction in cell viability of whole blood from the concentration of 10 μg/mL (59.17 μM) as measured by the tetrazolium-based colorimetric (MTT) assay (45). This discrepancy may be attributed to the different cell types used (isolated HMWB cells vs. whole blood culture), therefore the comparability of results is limited. Unlike glyphosate, all the three GBHs showed pronounced cell-killing activity at a high concentration range of 1,000 to 10,000 μM, especially in the WST-1 cell viability assay, regardless of the presence of the metabolic enzyme system and exposure durations. The increased cytotoxic potential of GBHs compared to pure glyphosate has been well-established and attributed to the presence of adjuvants in the formulations (13, 17, 29, 46–49). POEAs, the declared co-formulants in Glyfos, are reported to be over 1,000 times more cytotoxic than glyphosate alone (48, 49). Surprisingly, the different adjuvant content of the GBHs tested in this study did not considerably alter their cytotoxic effect, although the extent of cell death induced by Fozat 480 was less than that caused by the other two formulations, which can be explained by the lower concentration or toxicity of the other ingredients in Fozat 480. The adjuvant content of Fozat 480 (<5% hygroscopic substances) is not declared exactly in its MSDS; however, the dose-response relationship of cell viability suggests that it may also contain ethoxylated tallow amine surfactants, even if in lower concentration than the other two GBHs. This idea is supported by a study in which Fozat 480 exhibited a cytotoxicity pattern similar to other GBHs containing POEAs or ethoxylated ether alkylamine adjuvants (50). It is proven that ethoxylated adjuvants can disrupt cell membrane integrity and permeability, consequently increasing the bioavailability of glyphosate (51), but it is still not clear whether surfactants themselves are responsible for the cytotoxic effects or they interact with glyphosate synergistically. According to Wozniak et al. (30), genotoxicity detected in HMWB cells after exposure to technical glyphosate, Roundup 360 PLUS or the metabolite of glyphosate (aminomethylphosphonic acid, AMPA) was not the result of direct interaction of these compounds with the genetic material because no DNA adducts have been formed, rather due to effects mediated by reactive oxygen species (ROS) induced by the chemicals, which may also explain increased cell death. There is no clear evidence on the ROS-inducing potential of glyphosate (22, 25, 52); however, GBHs have been shown to induce oxidative stress (46, 53, 54), supporting the role of co-formulants in the cytotoxic effects observed in our experiments.

To avoid interference of cell death mechanism with genotoxic insults in our study, MN-inducing ability of the selected herbicides was investigated in a sub-cytotoxic (0–100 μM equivalent to glyphosate) concentration range. Technical glyphosate was able to produce a statistically significant increase of MN frequency in HMWB cells at the highest concentration of 100 μM after 20-h exposure in the absence and presence of S9. The observed lack of genotoxic effect in HMWB cells at lower concentrations and shorter exposure duration is in agreement with findings reported by Mladinic et al., who also did not observe a significant increase in the proportion of micronuclei in human lymphocytes in the same concentration range (22). By contrast, Santovito et al. found MN-inducing effect of glyphosate in human lymphocytes in a much lower concentration range of 0.0125–0.5 μg/mL (0.07–2.9 μM), but after a 48-h incubation period (37). Low glyphosate concentrations of 0.5, 2.91, and 3.5 μg/mL (2.9, 17.2, and 20.7 μM) were also able to induce a significant increase of MN frequency in human HepG2 cells after 4-h treatment in a study by Kasuba et al. (38); however, because HepG2 cells are cancerous human hepatocytes and are therefore characterized by an inherent genomic instability, MN results from these cells may not provide an adequate basis for comparison.

Unlike with glyphosate, a clear dose-dependent increase in MN frequency could be observed for all the three GBHs after both treatment regimens. Four-hour exposure to GBHs caused a statistically significant elevation of the MN frequency from 10 μM (Glyfos) and at 100 μM (Roundup Mega and Fozat 480), suggesting that co-formulants play a role not only in enhancing cell death but also in inducing genotoxic damage at non-cytotoxic concentrations. The more potent MN-inducing ability of Glyfos may be attributed to the POEA-content of this formulation, the direct DNA-damaging effect of which has already been established by previous toxicological studies (51, 52, 55, 56). Guilherme et al., exposing fish blood cells with Roundup formulation and its constituents, found that the genotoxic effect separately induced by POEA and glyphosate was not strengthened when the two substances were used in combination, ruling out a synergistic interaction between the ingredients of Roundup (52). Hao et al. demonstrated that POEA and Roundup exposure induce oxidative DNA lesions and other biochemical changes in human A549 cells, which were not detected in cells treated with glyphosate alone (14).

After 20-h exposure, although the difference in MN-inducing potential between the GBHs and glyphosate leveled out at 100 μM, Fozat 480 showed an increased genotoxic activity compared to other herbicides, as it already induced a statistically significant effect at 10 μM. This observation justifies our previous conclusion about the toxicity of non-declared adjuvant content of Fozat 480, which may be similar to that of Glyfos banned in Europe. Besides, exposure time seems to be an important factor in the clastogenic damage induced by glyphosate, as it could only have a significant effect after 20-h. This may be because glyphosate alone requires longer time to penetrate into cells and induce genomic damage, compared to being in a formulation where surfactant adjuvants would facilitate its entry by disrupting the cell membrane. This idea is partly corroborated by the study of Richard et al. that found glyphosate cytotoxicity increased with time (57), but contradicts with the report by Kasuba et al. who found significantly higher number of MN after 4-h than after 24-h in HepG2 cells exposed to glyphosate. However, they used metabolically active hepatocarcinoma-derived cell line that could result in the increased detoxification of glyphosate by 24-h (38). If the latter assumption is correct, we should have detected difference between the toxicity endpoints obtained with and without S9 treatment, especially after 20-h exposure; however, neither clear detoxification nor metabolic activation of any herbicides was observed in our study. Considering the lack of S9-dependent effects in the present study, we can conclude that metabolites have no increased clastogenic potential compared to the parent molecules. Finally, no significant changes in the proliferation index induced by both 4-h and 20-h exposure to all the tested herbicides could be detected, suggesting that neither toxicants interfere with mechanisms of cell division over the tested concentration range that confirms previous observations by Santovito et al. (37).

As with all studies, the current research also has certain limitations. First, the MN-inducing ability of co-formulants alone could not be measured due to limited information on the exact identity and concentration of the adjuvants, as well as other useful toxicity endpoints such as cell membrane permeabilization, mitochondrial potential, free radical levels, etc., were not examined, but may be the subject of future investigations. Second, our data exhibit some inter-experimental variability that may be due to the use of primary cell cultures obtained from blood samples of various donors. The different genetic background as well as lifestyle and environmental factors can strongly modify the susceptibility of individuals to genotoxic exposures and that variability is reflected in the variability of data between experiments. Increasing biological replicates could have reduced inter-experimental variability, and the validity of the results could have been improved by using machine-based automated MN scoring system. Third, CBMN assay is a well-established genotoxicity test but it has relatively low sensitivity and specificity to predict carcinogenicity, which has already been well-recognized (58–60); therefore, more sensitive, robust *in vitro* approaches with improved prediction of human carcinogenic risk may be needed. In addition, investigations with prolonged exposure times (>20-h) that better model realistic human exposure conditions may fill the knowledge gaps concerning chronic human health risks from accumulation of glyphosate in the food chain, because the presence of residual glyphosate in foodstuffs produced from glyphosate-treated crops, meat products from farmed animals that have consumed glyphosate-treated feed crops and contaminated drinking water constitute a continuous, albeit low-level, dietary exposure to the general population (61).

In conclusion, this is the first study that compares the MN-inducing potential of various glyphosate-based herbicides with their active ingredient in human peripheral white blood cells *in vitro*. Whilst glyphosate had a weak, but detectable genotoxic effect, GBHs exhibited both increased cytotoxic and genotoxic damage than the active substance that could be attributed to the effect of various adjuvants added to the formulations or to their interaction with glyphosate. Although some GBHs, that contain adjuvants with high toxicity, such as POEAs, have been banned in certain parts of the world (e.g., Glyfos), their counterparts with similar toxic properties are still on the market (e.g., Roundup Mega and Fozat 480). Thus, comprehensive toxicological assessment of co-formulants and complete formulations, together with the reconsideration of regulations allowing free access to GBHs are pressing challenges of the future. In addition, our findings underline the importance of biomonitoring studies based on micronucleus detection in order to minimize the consequent cancer risk in populations exposed to glyphosate-based herbicides.

## Data Availability Statement

The raw data supporting the conclusions of this article will be made available by the authors, without undue reservation.

## Ethics Statement

The studies involving human participants were reviewed and approved by Hungarian Ethical Committee for Medical Research. The patients/participants provided their written informed consent to participate in this study.

## Author Contributions

KN and BÁ designed the experiments. KN, IS, RA, TS, and AA performed the majority of toxicity studies. RA, TS, and AA carried out the microscopic analyses. KN, RA, TS, and AA evaluated the data and interpreted the results. KN, RA, and BÁ wrote and edited the manuscript. All authors contributed to the article and approved the submitted version.

## Conflict of Interest

The authors declare that the research was conducted in the absence of any commercial or financial relationships that could be construed as a potential conflict of interest.
