# Supplementary material for: Micronucleus Formation Induced by Glyphosate and Glyphosate-Based Herbicides in Human Peripheral White Blood Cells
Source: Front Public Health. 2021 May 24;9:639143. doi: 10.3389/fpubh.2021.639143 (PMC8180907; doi:10.3389/fpubh.2021.639143)
Supplement: Supplementary file 2 [file Table_2.docx]

**Supplementary table 2.** Frequency of binucleated cells with micronuclei (BNMN ‰) and proliferation index (PI) induced by 20-h exposure to sub-cytotoxic concentrations of glyphosate and GBHs with (+ S9) and without (- S9) metabolic activation system in human mononuclear white blood cells (MWBC) in cytokinesis-block micronucleus (CBMN) assay. Data are means of repeated experiments ± SEM.

|  | - S9 | | | |  | + S9 | | | |
| --- | --- | --- | --- | --- | --- | --- | --- | --- | --- |
| Concentration (µM) | Glyphosate | Roundup Mega | Fozat 480 | Glyfos |  | Glyphosate | Roundup Mega | Fozat 480 | Glyfos |
|  | **BNMN ‰ ± SEM** | | | | | | | | |
| 0 | 1.88 ± 0.44 | | | |  | 2.33 ± 0.49 | | | |
| 0.1 | 4.25 ± 0.52 | 4.61 ± 1.17 | 3.97 ± 0.55 | 2.94 ± 0.33 |  | 3.68 ± 0.36 | 4.53 ± 0.54 | 4.51 ± 0.63 | 3.75 ± 0.59 |
| 1 | 3.88 ± 0.45 | 5.51 ± 1.74 | 5.95 ± 0.42 | 4.57 ± 1.17 |  | 3.64 ± 0.36 | 5.50 ± 1.69 | 7.03 ± 1.19 | 4.20 ± 1.00 |
| 10 | 6.63 ± 0.67 | 5.89 ± 1.84 | 9.13 ± 1.52* | 6.43 ± 1.73 |  | 6.13 ± 0.66 | 6.05 ± 1.91 | 8.59 ± 2.11* | 7.01 ± 1.57 |
| 100 | 8.69 ± 2.34** | 10.12 ± 1.98* | 10.43 ± 0.59*** | 10.30 ± 2.92** |  | 9.49 ± 1.07*** | 7.62 ± 2.31* | 10.00 ± 1.81* | 9.87 ± 3.04* |
| BLEO | 34.74 ± 0.55*** | | | |  | 25.49 ± 0.85*** | | | |
|  | **PI ± SEM** | | | | | | | | |
| 0 | 1.60 ± 0.06 | | | |  | 1.71 ± 0.06 | | | |
| 0.1 | 1.67 ± 0.16 | 1.49 ± 0.09 | 1.45 ± 0.06 | 1.53 ± 0.15 |  | 1.42 ± 0.03 | 1.55 ± 0.15 | 1.54 ± 0.08 | 1.52 ± 0.18 |
| 1 | 1.55 ± 0.17 | 1.62 ± 0.01 | 1.49 ± 0.09 | 1.60 ± 0.17 |  | 1.59 ± 0.11 | 1.65 ± 0.18 | 1.48 ± 0.09 | 1.40 ± 0.10 |
| 10 | 1.52 ± 0.09 | 1.67 ± 0.12 | 1.52 ± 0.13 | 1.47 ± 0.12 |  | 1.43 ± 0.02 | 1.46 ± 0.15 | 1.42 ± 0.11 | 1.39 ± 0.10 |
| 100 | 1.42 ± 0.04 | 1.48 ± 0.06 | 1.53 ± 0.14 | 1.32 ± 0.07 |  | 1.51 ± 0.11 | 1.49 ± 0.08 | 1.37 ± 0.02 | 1.39 ± 0.13 |
| BLEO | 1.49 ± 0.04 | | | |  | 1.50 ± 0.08 | | | |

Statistically significant (* p < 0.05, ** p < 0.01, ***p < 0.001) increase was determined by comparing the frequency of binucleated cells with micronuclei or proliferation index induced by various doses of test chemicals to the background level of untreated cells by ANOVA with Dunnett’s post hoc test.

Difference in the frequency of binucleated cells with micronuclei or proliferation index between S9-treated and S9-untreated cells induced by the same concentration of glyphosate and GBHs was tested by ANOVA with Dunnett’s post hoc test.

BLEO: 1.3 µM bleomycin sulfate as positive control
